# An invasion deep into the nucleus: stripe rust effector Pst21674 inhibits transcription factor TaASR3 in wheat

**DOI:** 10.1093/plphys/kiad542

**Published:** 2023-10-14

**Authors:** Yee-Shan Ku

**Affiliations:** Assistant Features Editor, Plant Physiology, American Society of Plant Biologists; School of Life Sciences and Centre for Soybean Research of the State Key Laboratory of Agrobiotechnology, The Chinese University of Hong Kong, Hong Kong SAR, China

In the natural environment, plants are constantly challenged by pathogens, including bacteria and fungi. Plants have evolved defenses against pathogens, and pathogens have evolved strategies, including the production of effectors, through which to suppress plant defenses. Understanding the interplay between plant defenses and pathogen effectors is important for designing strategies to promote plant immunity.

Pathogenic effectors can be proteins, metabolites, or small RNA molecules (Jaswal et al. 2020). Fungal effectors are typically secreted from the fungal haustorium and therefore include a signal peptide at their N terminus. They also frequently include organelle localization signals to mediate the proper subcellular localization in the host cell (e.g. nucleus, mitochondria). Although effector proteins have long been shown to be essential for pathogenicity, the mechanism behind their action is still unclear.

*Puccinia striiformis* f. sp. *tritici* (*Pst*) is the causal agent of wheat stripe rust. In this issue of *Plant Physiology*, Zheng et al. (Zheng et al. 2023) investigated the mechanism of action of Pst21674, previously identified as a haustorium-secreted protein that interfered with plant defenses. Pst21674 has an N-terminal signal sequence and a C-terminal nuclear-localization signal.

The authors first used a yeast-two-hybrid library to screen for wheat proteins that interact with Pst21674. Subcellular localization, *in vitro* pulldown, and co-immunoprecipitation assays revealed that Pst21674 interacts with a wheat transcription factor, TaASR3, in the nucleus of the host cell (Zheng et al. 2023). The authors next carried out a series of assays to demonstrate the functional roles of Pst21674 and TaASR3 in the infection. The authors showed that host-induced gene silencing of *Pst21674* reduced the virulence of *Pst*. The positive role of TaASR3 in immunity was demonstrated by the enhanced resistance when *TaASR3* was overexpressed in wheat and enhanced susceptibility when it was silenced.

RNA-seq was employed to search for genes regulated by TaASR3. Overexpression of *TaASR3* was associated with the elevated expressions of defense-related genes. However, when *Pst21674* was coexpressed with *TaASR3*, the expressions of these defense-related genes were suppressed. The authors also investigated the mechanism behind the inhibitory effect of Pst21674 on TaASR3. Size exclusion chromatography, bimolecular fluorescence complementation, and luciferase complementation imaging revealed that TaASR3 proteins form a functional tetramer. However, when *Pst21674* was coexpressed with *TaASR3*, self-multimerization of TaASR3 proteins was reduced. Such a reduction of self-multimerization was coupled with the suppressed expressions of defense-related genes. Based on these results, the authors concluded that TaASR3 proteins form a functional tetramer to promote the expressions of defense-related genes. However, upon the invasion of Pst21674 into the nucleus, the self-multimerization of TaASR3 proteins is reduced. As a result, the abundance of functional TaASR3 tetramer and the expressions of the target defense-related genes are suppressed.

This study opens possibilities for future research. For example, the characterization of the defense-related genes regulated by TaASR3 and the functional roles of these genes will improve the understanding of host immunity in wheat, and understanding the pathogenic mechanism of Pst21674 could point to strategies to promote the host immunity, including by expression of antisense *Pst21647* RNA in the host wheat to silence *Pst21647* and promote resistance to *Pst* (Zheng et al. 2023).

**Figure 1.**
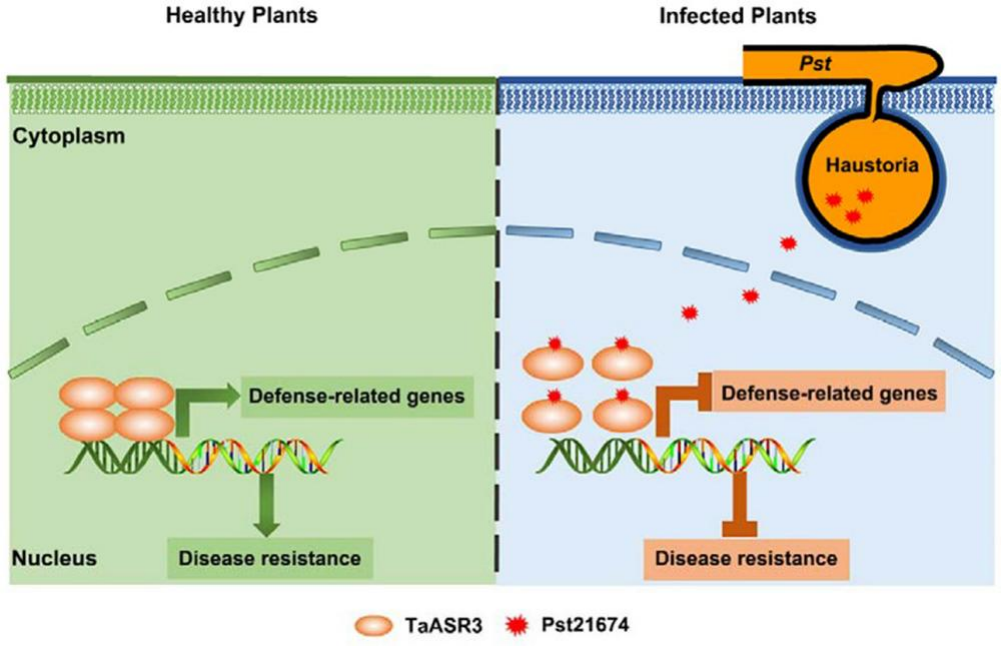
Upon the infection by *Pst*, the effector protein Pst21674 is secreted and enters the nucleus of the host cell, where it inhibits the transcriptional activity of TaASR3. This inhibition prevents induction of defense-related genes and results in the virulence. This figure is adopted from Zheng et al. 2023 (Zheng et al. 2023).

## Dive Curated Terms

The following phenotypic, genotypic, and functional terms are of significance to the work described in this paper:

nucleus CHEBI: CHEBI:33252
